# Prenatal Alcohol Exposure Predicts Academic Outcomes from Childhood to Adolescence: A Prospective Longitudinal Study Based on Meconium Ethyl Glucuronide

**DOI:** 10.3390/bs16050715

**Published:** 2026-05-06

**Authors:** Stefan Mestermann, Maike Broich, Peter A. Fasching, Matthias W. Beckmann, Oliver Kratz, Jonas Hemetsberger, Maximilian Bailer, Bernd Lenz, Johannes Kornhuber, Anna Eichler, the IMAC-Mind-Consortium

**Affiliations:** 1 Department of Child and Adolescent Mental Health, University Hospital Erlangen, Friedrich-Alexander-Universität Erlangen-Nürnberg (FAU), 91054 Erlangen, Germanyanna.eichler@uk-erlangen.de (A.E.); 2 Department of Obstetrics and Gynaecology, University Hospital Erlangen, Friedrich-Alexander-Universität Erlangen-Nürnberg (FAU), 91054 Erlangen, Germany; 3 Department of Psychiatry and Psychotherapy, University Hospital Erlangen, Friedrich-Alexander-Universität Erlangen-Nürnberg (FAU), 91054 Erlangen, Germany; 4 Department of Addictive Behavior and Addiction Medicine, Central Institute of Mental Health, Medical Faculty Mannheim, Heidelberg University, 68159 Mannheim, Germany; 5 University Medical Center Hamburg-Eppendorf, 20251 Hamburg, Germany

**Keywords:** prenatal alcohol exposure, ethyl glucuronide, academic achievement, school grades, educational track, fetal alcohol spectrum disorder, perinatal period, neurodevelopment

## Abstract

Background: Prenatal alcohol exposure (PAE) is a major preventable cause of neurodevelopmental impairment. Little is known about its impact on real-life child/adolescent academic outcomes, which are key functional indicators of long-term social participation. Methods: PAE was assessed in a sample of 156 participants via (1) meconium ethyl glucuronide (EtG) concentration (≥10 ng/g; EtG-positive, *n* = 36) and (2) third-trimester maternal self-report (SR-positive, *n* = 40). At primary school (6–10 years, t1) and secondary school ages (12–14 years, t2), exposed and non-exposed children were compared regarding primary school grades (t1) and secondary track placement (t2). Results: At t1, EtG-positive children exhibited lower school report grades by trend (*n* = 15, *p* = 0.068–0.085) with a moderate effect size (*r* = 0.36–0.39). SR-positive children demonstrated significantly lower school report grades (*n* = 15, *p* = 0.016) with strong effect (*r* = 0.56). At t2, both exposure groups had increased trend-significant odds of attending lower educational tracks (EtG-positive/-negative: *n* = 153, OR = 1.84, *p* = 0.059; SR-positive/-negative: *n* = 153, OR = 1.78, *p* = 0.066). Conclusions: PAE is associated with reduced real-life academic outcomes across development. School performance represents a sensitive functional outcome of neurodevelopmental vulnerability following PAE. Meconium EtG may contribute to postnatal identification of affected children.

## 1. Introduction

Prenatal alcohol exposure (PAE) affects approximately one in ten pregnancies worldwide, with particularly high prevalence in Europe (Popva et al., 2017). Alcohol is a well-established teratogen that interferes with central nervous system (CNS) maturation, giving rise to a spectrum of structural and functional abnormalities summarized as fetal alcohol spectrum disorders (FASDs) (Mattson et al., 2019).

Neurocognitive impairments associated with PAE include deficits in executive functioning, working memory, attention regulation and learning processes (Chu et al., 2022; Jacobson et al., 2021). These domains are directly linked to academic competence. Meta-analytic evidence demonstrated robust associations between executive functions and academic outcomes in childhood (Spiegel et al., 2021), and broader cognitive and personality traits have been shown to predict school performance longitudinally (Demetriou et al., 2023).

Studies examining children with PAE consistently report lower scores in standardized reading and mathematics assessments (Glass et al., 2017; O’Leary et al., 2013; Schneider et al., 2024). However, fewer studies have examined real-life indicators, such as school report grades or secondary school track placement decisions (Alati et al., 2013). These real-life measures reflect classroom performance and teacher-based evaluation, providing clinically meaningful insight into everyday functional impairment. Given that educational attainment significantly impacts socioeconomic status and health trajectories across the lifespan, early identification of biological risk markers is crucial. Additionally to school report grades, school tracking decisions represent meaningful long-term academic outcomes (Alati et al., 2013; O’Leary et al., 2013).

Yet, assessment of PAE remains challenging. Maternal self-report is vulnerable to recall inaccuracies and social desirability bias, particularly in stigmatized contexts such as alcohol use during pregnancy (Eichler et al., 2016; Mestermann et al., 2023). Meconium biomarkers—especially ethyl glucuronide (EtG)—have emerged as objective indicators of third-trimester alcohol exposure. Prior studies have linked EtG or fatty acid ethyl esters (FAEEs) to cognitive, behavioral, inflammatory and neurodevelopmental outcomes in childhood and adolescence (Eichler et al., 2018; Maschke et al., 2021a; Min et al., 2015; Roetner et al., 2023; Singer et al., 2021) and facial phenotypes (Maschke et al., 2021b), as well as, most recently, as potential predictors of PAE-induced poor language development (Min et al., 2026). Nevertheless, evidence regarding long-term academic functioning remains scarce. The present study addresses this gap by examining whether biologically verified and self-reported PAEs are associated with school report grades in primary school and academic level in adolescence. Two aims were pursued:

1. To investigate the relationship between PAE and real-world academic outcomes across development: Are there differences in school report grades in primary school (i.e., school grades) and academic level in secondary school (i.e., educational track) between children prenatally exposed to alcohol and those not exposed, based on meconium EtG concentration and maternal prenatal self-report?
2. To further evaluate meconium EtG as an early biomarker of clinically meaningful neurodevelopmental consequences: Are the expected associations significant, and can EtG serve as a valid biomarker for intrauterine alcohol exposure?

## 2. Materials and Methods

### 2.1. Study Design

Data derive from the Franconian Cognition and Emotion Studies (FRANCES), a longitudinal follow-up of the Franconian Maternal Health Evaluation Study (FRAMES) (Eichler et al., 2016; Eichler et al., 2018; Roetner et al., 2023). This cohort investigates consequences of prenatal risk exposures—including alcohol consumption—on offspring development. Recruitment occurred during the third trimester (t0; 2005–2007) at the Department of Obstetrics and Gynaecology, University Hospital Erlangen (pregnant women ≥18 years, ≥30 weeks of gestation). Follow-ups were conducted at primary school age (t1; 2012–2015) and early adolescence (t2; 2019–2021).

At t1, a random subsample of *n* = 501 out of *n* = 1100 women from t0 was contacted due to limited study capacity. The women chosen at random were no different from the women not chosen at random in terms of whether they had graduated from high school (yes/no, χ^2^(1) = 0.122, *p* = 0.726), their marital status (married yes/no, χ^2^(1) = 0.453, *p* = 0.501), or their family’s income (<2000 euros per month, 2000–4000 euros per month, >4000 euros per month: χ^2^(2) = 3.41, *p* = 0.182). Additional targeted recruitment of *n* = 117 women was performed to increase representation of children at risk. Compared to the *n* = 482 women who were not selected, the women with a specific risk background (prenatal alcohol/nicotine or prenatal depression) were less likely to have a high school diploma (χ^2^(1) = 8.03, *p* = 0.005) and had a lower family income (χ^2^(2) = 11.0, *p* = 0.004). However, there was no difference in marriage between the groups (χ^2^(1) = 0.52, *p* = 0.473). A total of *n* = 245 mother–child dyads participated (mean child age 7.74 years). At t2, 186 adolescents (75.9%) were reassessed (mean age 13.3 years).

### 2.2. Sample

After exclusion of twin pairs (*n* = 6) and missing EtG data (*n* = 45), *n* = 197 dyads were available for t1 and *n* = 153 for t2 analysis. Valid school report data at t1 were available for *n* = 15 children (7.6%; the sample size was small because children in Germany do not receive report cards with grades until the end of the second year of school; the majority of children were in their first year of school, 56.2%, or the first semester of their second year, 34.0%, at t1), valid academic level data at t2 were available for *n* = 153 children (100%), with *n* = 12 children having both measures. Thus, the present complete sample comprises *n* = 156 participants. For these *n* = 156, at t1, there were *n* = 36 EtG+/*n* = 40 SR+ children, and at t2, there were *n* = 35 EtG+/*n* = 38 SR+ children included. The study was approved by the Local Ethics Committee (Nr. 3374 and Nr. 4596) and was conducted in accordance with the Declaration of Helsinki. All adult participants gave informed consent, all minors informed assent. See Table 1 for sample characteristics.

### 2.3. Data Assessment

Socioeconomic Status (SES)

Socioeconomic status (SES) was calculated at t1 based on maternal and paternal educational level (4 levels: <9, 9, 10 or 13 years of school attendance) and net family income (6 levels: <1000 to >5000 Euro) (sum index: 2 × educational level + 1 × net family income level, theoretical range: 3–14)

PAE

Meconium ethyl glucuronide: Meconium samples were collected within 2–24 h postpartum and analyzed according to established neurochemical procedures. A cut-off of ≥10 ng/g defined prenatal alcohol exposure (EtG-positive), consistent with validation studies (Bakdash et al., 2010; Himes et al., 2015).

Maternal self-report: Alcohol consumption during pregnancy was assessed via a Likert-type item during third-trimester interviews (Eichler et al., 2016). Responses were grouped into two dichotomous categories: PAE-negative (SR-negative, including responses of “I don’t drink in general”, *n* = 13, 8.3%, and “I didn’t drink during pregnancy”, *n* = 103, 66.0%) or PAE-positive (SR-positive, including responses of “I rarely drank during pregnancy”, *n* = 39, 25%, and “I drank one or more glasses per day during pregnancy”, *n* = 1, 0.6%).

Academic Outcomes

To assess primary school report grades (t1), official school report grades (1 = best; 6 = worst) were obtained for the following subjects in German schools: Mathematics, German and General Studies (local history, geography and biology). Composite mean scores were calculated: 1. Main subject score (Mathematics, German) and 2. Overall school performance score (Mathematics, German, General Studies). For the assessment of secondary school academic level (t2), educational track was categorized as: Higher level (>12 years of planned school attendance); Lower/Medium level (≤12 years of planned school attendance).

**Figure 1:**
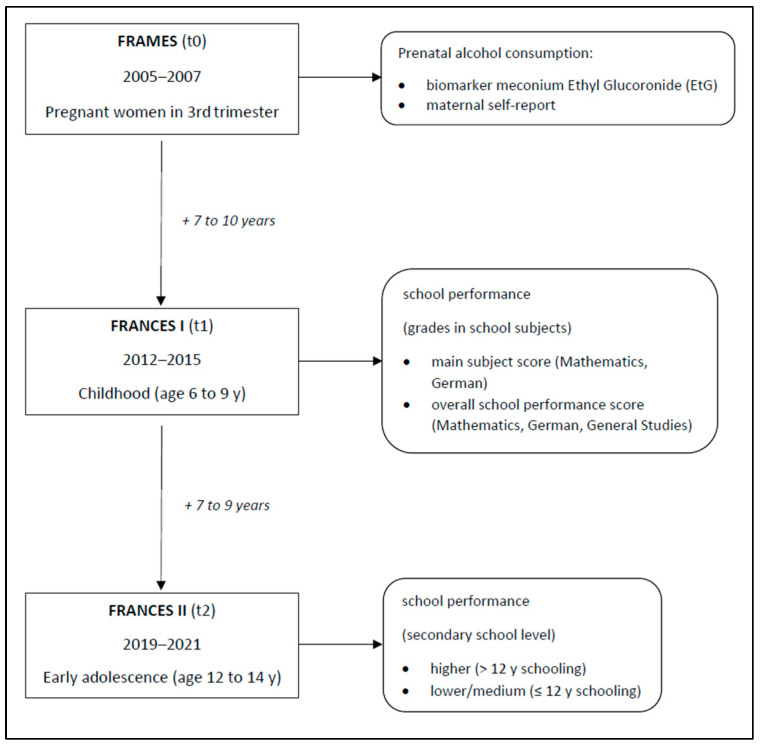
Study design and assessed parameters. Notes: EtG: ethyl glucuronide, y: years.

Study design and assessed parameters are depicted in Figure 1.

### 2.4. Statistical Analyses

Analyses were performed using IBM^®^ SPSS^®^ 24.0. Descriptive data were reported as means (M) with standard deviation (SD) or frequencies (n) with percentages (%). Non-parametric Wilcoxon U tests were performed to measure the differences between the EtG-positive/negative and SR-positive/negative groups in primary school grades, due to the ordinal-scaled, non-normally distributed data. Secondary school level frequencies between exposed and non-exposed groups were compared using chi-square tests. Significance level was defined as *p* < 0.05 (one-tailed). Effect sizes were calculated by |r| (Wilcoxon U test), interpreted as weak (0.1–0.3), moderate (0.3–0.5), and strong (>0.5), and by odds ratios (ORs, chi-square tests). There were no covariates that we controlled for due to the small sample size and because of the following—largely lacking—associations: SES was not different between EtG+/− or SR+/− groups (Table 1). School performance data at t1 (Main subject score: *r* = 0.038, *p* = 0.893; Overall school performance: *r* = 0.006, *p* = 0.982) were not associated with SES; at t2 SES was higher in the higher school level group (t(151) = −2.99, *p* = 0.003). School performance data at t1 (Main subject score: t(13) = −0.50, *p* = 0.627; Overall school performance: t(13) = −0.61, *p* = 0.551) or at t2 (χ^2^(1) = 0.61, *p* = 0.436) were not different between sexes.

## 3. Results

In the sample of *n* = 156 children, *n* = 36 had meconium EtG levels above the 10 ng/g cut-off (23.1%), and *n* = 40 of the mothers reported prenatal alcohol consumption (25.6%). As illustrated in Table 2, the correlation between the two groups was significant (χ^2^ = 4.31, *p* = 0.038) at low effect size (Φ = 0.166). Additionally, 78.3% of EtG− children had mothers who declined prenatal alcohol consumption, while 22 mothers with EtG+ children did not report prenatal consumption (61.1% of EtG+ cases). In numbers, *n* = 14 (8.97%) of the mothers both reported prenatal alcohol consumption (SR*) and had children with positive EtG levels in meconium (EtG+).

Within the *n* = 36 EtG+ group, EtG values ranged from 10 ng/g to 2400 ng/g (M = 76.5, SD = 281). EtG level and school performance at t1 were not significantly correlated (Main subject score: *r* = 0.264, *p* = 3.42; Overall school performance: *r* = 0.237, *p* = 0.394). At t2 EtG levels were significantly lower in children with higher academic level (M = 38.0, SD = 114) than in children with lower academic level (M = 148, SD = 449; t(151) = 1.748, *p* = 0.043).

### 3.1. Primary School Performance (t1)

**Figure 2:**
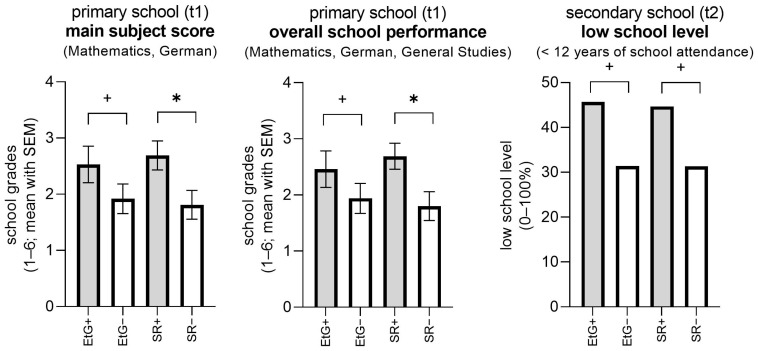
School performance differences in EtG+/− and SR+/− groups. EtG: ethyl glucuronide. EtG−: negative EtG in meconium (<10 ng/g). EtG+: positive EtG in meconium (≥10 ng/g). SR: self-report, SR+/−: positive/negative self-report for prenatal alcohol consumption. Average grades (1–6, 1 = best, 6 = worst). High: ≥12 years of school attendance, low: <12 years of school attendance, * *p* < 0.05 (one-tailed), ^+^ *p* < 0.10 (one-tailed).

With a moderate effect size (0.36/0.39), children who tested positive for EtG exhibited lower grades, with statistical trends (*p* = 0.085/0.068) (see Table 3, Figure 2). There were no significant differences for specified educational domains (Mathematics *p* = 0.203, German *p* = 0.204, General Studies *p* = 0.449), and the integrated total scores proved to be the most significant.

SR-positive children demonstrated significantly lower grades across subjects (*p* = 0.018/0.018) with strong effect sizes (*r* = 0.56/0.56) (see Table 4).

### 3.2. Secondary School Performance (t2)

EtG-positive adolescents exhibited an elevated probability of pursuing lower educational tracks, a finding that was significant by trend (OR = 1.84, *p* = 0.059); the risk of lower track placement for children who were exposed was 1.84 times higher than for children who were not exposed. Adolescents who were SR-positive demonstrated comparable risk levels that were significant by trend (OR = 1.78, *p* = 0.066); the risk of lower track placement for children who were exposed was 1.78 times higher than for children who were not exposed (Table 5, Figure 2).

## 4. Discussion

The present longitudinal findings indicate that PAE is associated with measurable impairments in everyday academic functioning from primary school into adolescence. From a clinical developmental perspective, these results are highly relevant, as academic performance represents a functional endpoint of CNS integrity and adaptive capacity.

Neurodevelopmental alterations associated with PAE are well documented and encompass deficits in executive functioning, attentional control, working memory, processing speed, and verbal learning (Chu et al., 2022; Jacobson et al., 2021; Mattson et al., 2019). These domains form the cognitive substrate required for structured classroom learning, sustained task engagement, and cumulative knowledge acquisition. Even subtle alterations in these systems may remain clinically underrecognized in early childhood but become increasingly evident once formal academic demands intensify.

Importantly, the present study extends prior work based on standardized testing (Glass et al., 2017; O’Leary et al., 2013) by demonstrating associations with real-life school outcomes. School report grades reflect continuous performance across multiple contexts, including classroom behavior, homework completion, language expression, and self-regulation. From a child and adolescent psychiatric/pediatric clinical standpoint, such real-world indicators may be more informative than isolated neuropsychological test scores when evaluating long-term functional impact.

The observed tendency toward lower secondary school track placement among alcohol-exposed adolescents further underlines the potential persistence of these vulnerabilities. In structured educational systems with early academic tracking, minor performance differences during primary school may translate into long-term educational stratification. Clinically, this highlights the importance of early developmental surveillance in children with known or suspected PAE.

Both exposure indicators showed consistent directional associations, and maternal self-report yielded statistically stronger findings. This underlines the indispensability of subjective maternal SR as the most widely used method for PAE assessment in clinical practice (McQuire et al., 2016). Yet, it is well established that alcohol consumption during pregnancy is frequently underreported due to recall bias and stigma (Eichler et al., 2016; Mestermann et al., 2023). Meconium EtG provides an objective marker of third-trimester exposure and has previously been linked to cognitive and behavioral outcomes (Eichler et al., 2018; Maschke et al., 2021b; Roetner et al., 2023). The present results suggest that EtG may also carry prognostic value regarding later academic functioning. The slightly higher decline of PAE vs. non-PAE performance scores at t1 in the SR group might reflect a higher sociodemographic bias and/or risk factors in the SR group, such as lower SES. t1 results in the EtG group yielded moderate effect sizes and trend significance, presumably due to the limited number of participants. It is possible that replication studies with a greater sample size might demonstrate statistical significance.

From a clinical implementation perspective, these findings support several considerations:

- Children with documented PAE—whether via biomarker or clinical history—should receive structured developmental follow-up extending into school age.
- Even in the absence of dysmorphic features or global intellectual disability, subtle neurocognitive vulnerabilities may manifest in academic underperformance, which is consistent with a diagnosis of alcohol-related neurodevelopmental disorders (ARNDs) and/or neurobehavioral disorder associated with PAE (ND-PAE).
- Collaboration between pediatricians, child and adolescent psychiatrists and psychologists, and educational professionals is essential to ensure early support measures.

Given that educational attainment is (among other factors) strongly associated with mental health (Monzonís-Carda et al., 2025), employment opportunities, and long-term socioeconomic stability (Pensiero & Barone, 2023), academic difficulties may represent an early mediator of cumulative disadvantage. Prevention of PAE remains paramount; however, secondary prevention through early detection and educational support may mitigate long-term sequelae.

The present data are subject to certain limitations. The small number of available school reports at t1 limits statistical power. Maternal self-report did not quantify exact timing and dosage of alcohol use. School performance was not measured consistently for t1 and t2. Future studies should include larger samples, more detailed exposure and more consistent school performance assessments.

## 5. Conclusions

This longitudinal study demonstrates that PAE is associated with lower academic achievement in primary school and shows a tendency toward less academically demanding educational pathways in adolescence. Importantly, these associations were observed in a community-based cohort not limited to clinically diagnosed FASD or other psychiatric disorders, indicating that subclinical exposure levels may still carry developmental relevance. Child/adolescent psychiatric and pediatric care providers should therefore incorporate systematic developmental monitoring for exposed children, particularly during the transition to formal schooling. Meconium EtG appears to offer added value as an objective early life biomarker that may support risk stratification.

## Figures and Tables

**Table 1:** Sample characteristics of participating children (M (SD)/N (%)) and subgroup differences.

		Total Sample	EtG+	EtG−	*t*(df)/*χ*^2^(df)	SR+	SR−	*t*(df)/*χ*^2^(df)
T1 (*n* = 15)								
Child sex	m	6 (40.0)	4 (66.7)	2 (33.3)	2.96 ^+^ (1)	3 (50.0)	3 (50.0)	0.42 (1)
	w	9 (60.0)	2 (22.2)	7 (77.8)		3 (33.3)	6 (66.7)	
Child age [y]		8.55 (0.65)	8.83 (0.42)	8.36 (0.73)	−1.42 (13)	8.53 (0.70)	8.57 (0.66)	0.104 (13)
SES		11.67 (2.02)	11.33 (2.34)	11.89 (1.90)	0.51 (13)	11.00 (2.53)	12.11 (1.62)	1.05 (13)
Grade	2	5 (33.3)	1 (20.0)	4 (80.0)	1.75 (2)	2 (40.0)	3 (60.0)	0.08 (2)
	3	7 (46.7)	3 (42.9)	4 (57.1)		3 (42.9)	4 (57.1)	
	4	3 (20.0)	2 (66.7)	1 (33.3)		1 (33.3)	2 (66.7)	
T2 (*n* = 153)								
Child sex	m	80 (52.3)	17 (21.2)	63 (78.8)	0.251 (1)	24 (30.0)	56 (70.0)	2.40 (1)
	w	73 (47.7)	18 (24.7)	55 (75.3)		14 (19.2)	59 (80.8)	
Child age [y]		13.31 (0.34)	13.39 (0.39)	13.29 (0.33)	−1.66 (151)	13.30 (0.37)	13.31 (0.34)	0.26 (151)
SES		11.42 (2.13)	11.60 (1.83)	11.37 (2.21)	−0.55 (151)	11.76 (2.05)	11.31 (2.15)	0.25 (151)
School level (in years of attendance)	>12	100 (65.4)	19 (19.0)	81 (81.0)	2.46 (1)	21 (21.0)	79 (79.0)	2.28 (1)
	≤12	53 (34.6)	16 (30.2)	37 (69.8)		17 (32.1)	36 (67.9)	

Notes: EtG: ethyl glucuronide. EtG−: meconium EtG < 10 ng/g. EtG+: meconium EtG in ≥10 ng/g. SR+/−: positive/negative third-trimester self-report on alcohol consumption during pregnancy. SES: Socioeconomic status (sum index out of maternal and paternal educational level and family income) y: years, ^+^ *p* < 0.10 (two-tailed).

**Table 2:** Meconium EtG above the cut-off and positive self-reports on prenatal alcohol consumption.

	SR−	SR+	*χ* ^2^	*p*	Φ
EtG−	94	26	3.68 (1)	0.055 ^+^	0.155
EtG+	22	14			

Notes: EtG: ethyl glucuronide. EtG−: negative EtG in meconium (<10 ng/g). EtG+: positive EtG in meconium (≥10 ng/g). SR: self-report, SR+/−: positive/negative self-report for prenatal alcohol consumption, ^+^ *p* < 0.10 (two-tailed).

**Table 3:** School report grades at t1 in EtG+/− groups.

	EtG−		EtG+		Z	*p*	*r*
	M (SD)	Range	M (SD)	Range			
	*n* = 9		*n* = 6				
Main subject score	1.92 (0.79)	1.00–3.00	2.53 (0.80)	1.50–3.50	−1.50	0.068 ^+^	0.39
Overall school performance	1.94 (0.80)	1.00–3.00	2.46 (0.80)	1.33–3.33	−1.38	0.085 ^+^	0.36

Notes: EtG: ethyl glucuronide. EtG−: negative EtG in meconium (<10 ng/g). EtG+: positive EtG in meconium (≥10 ng/g). Average grades (1–6, 1 = best, 6 = worst). M: mean, SD: standard deviation, *U*-Tests (*Z*-Score) with test effect size *r*, ^+^ *p* < 0.10 (one-tailed).

**Table 4:** School report grades at t1 in SR+/− groups.

	SR−		SR+		Z	*p*	*r*
	M (SD)	Range	M (SD)	Range			
	*n* = 9		*n* = 6				
Main subject score	1.81 (0.77)	1.00–3.00	2.69 (0.63)	2.00–3.50	−2.15	0.016 *	0.56
Overall school performance	1.80 (0.77)	1.00–3.00	2.69 (0.57)	2.00–3.33	−2.15	0.016 *	0.56

Notes: SR: self-report, SR+/−: positive/negative self-report for prenatal alcohol consumption. Average grades (1–6, 1 = best, 6 = worst). M: mean, SD: standard deviation, *U* Tests (*Z*-Score) with test effect size *r*, * *p* < 0.05 (one-tailed).

**Table 5:** School level at t2 in the EtG+/− and SR+/− groups.

	EtG−	EtG+	*χ*^2^ (df)	*p*	OR	SR−	SR+	*χ*^2^ (df)	*p*	OR
	*n* (%)	*n* (%)				*n* (%)	*n* (%)			
High	81 (68.6)	19 (54.3)	2.46 (1)	0.059 ^+^	1.84	79 (68.7)	21 (55.3)	2.28 (1)	0.066 ^+^	1.78
Low	37 (31.4)	16 (45.7)				36 (31.3)	17 (44.7)			

Notes: High: ≥12 years of school attendance, low: <12 years of school attendance, SR: self-report, SR+/−: positive/negative self-report for prenatal alcohol consumption, Chi-square test with OR: Odds Ratio, ^+^ *p* < 0.10 (one-tailed).

## Data Availability

The datasets generated during and/or analyzed during the current study are available from the corresponding author on reasonable request.

## Abbreviations

The following abbreviations are used in this manuscript:

ARNDs	alcohol-related neurodevelopmental disorders
CNS	central nervous system
EtG	ethyl glucuronide
FASDs	fetal alcohol spectrum disorders
FRAMES	Franconian Maternal Health Evaluation Study
FRANCES	Franconian Cognition and Emotion Studies
ND-PAE	neurobehavioral disorder associated with prenatal alcohol exposure
PAE	prenatal alcohol exposure
SES	socioeconomic status
SR	self-report
